# The effect of intraperitoneal chemotherapy on early pain hyperalgesia in patients following elective laparoscopic transabdominal resection of rectal cancer

**DOI:** 10.18632/oncotarget.18417

**Published:** 2017-06-08

**Authors:** Min Liang, Chang-Ying Li, Chun-Guang Ren, Zong-Wang Zhang, Zhi-Jian Fu

**Affiliations:** ^1^ Department of Pain Management, Shandong Provincial Hospital Affiliated to Shandong University, Jinan, Shandong, P.R. China; ^2^ Department of Anesthesiology, Liaocheng People’s Hospital, Liaocheng, Shandong, P.R. China

**Keywords:** chemotherapy, hyperalgesia, lobaplatin, colorectal cancer, sufentanil

## Abstract

**Background:**

Chemotherapy has been associated with hyperalgesia. This prospective study was designed to investigate the effect of intraperitoneal chemotherapy with lobaplatin on post-operative pain intensity and sufentanil requirements after laparoscopic transabdominal resection of rectal cancer.

**Methods:**

Eighty subjects (40 subjects treated with intraperitoneal chemotherapy and 40 subjects without chemotherapy treatment) scheduled for laparoscopic transabdominal resection of rectal cancer were included in this study. All subjects received standardized anesthetic and patient-controlled analgesia using sufentanil for 72 h post-surgery, as the only analgesics. Pain intensity scores, cumulative sufentanil requirements and side effects were recorded until 72 h post-surgery.

**Results:**

Following intraperitoneal chemotherapy, patients had a significantly higher total post-operative sufentanil requirement (193 μg *vs*. 142 μg; *P* = 0.008), significantly higher verbal rating scale post-surgery pain intensity scores at rest and with coughing (*P* < 0.05), and a significantly worse functional activity score (*P* < 0.05) over 72 h, compared with those without intraperitoneal chemotherapy. There were no post-operative differences in the incidence of side-effects (post-operative nausea [*P* = 0.189], vomiting [*P* = 0.311], pruritus [*P* = 0.263], respiratory depression [*P* = 1.000], and dizziness [*P* = 0.712]) between the two groups.

**Conclusion:**

Intraperitoneal chemotherapy is associated with significantly increased post-operative sufentanil requirements and pain intensity, suggesting chemotherapy-associated hyperalgesia.

## BACKGROUND

It is estimated that 20% to 40% of patients with cancer who receive chemotherapy will develop painful chemotherapy-induced peripheral neuropathy (CIPN), which commonly occurs in patients using taxanes, platinums and vinca alkaloids [1–3]. The symptoms of CIPN are sensory and manifest mainly as hyperalgesia, allodynia, tingling and numbness [4]. CIPN can persist from months to years beyond chemotherapy completion, significantly reducing the quality of life of cancer patients and the efficacy of chemotherapy [5–9]. It is difficult to manage, and a variety of drugs with diverse mechanisms have not been effective in treating the condition [10]. It has been demonstrated that lobaplatin, a third-generation platinum antitumor agent, could significantly inhibit the growth of colorectal carcinoma cells in vitro and induce the apoptosis of LOVO cells [11]. Previous clinical trials have indicated that lobaplatin has antitumor effects in human solid tumors, such as ovarian cancer, breast cancer and colorectal cancer [12–14]. Intraperitoneal chemotherapy (IPC) with lobaplatin has been shown to be a practicable procedure with acceptable safety and can prolong the overall survival of patients with peritoneal carcinomatosis (PC) and in patients with gastrointestinal malignancies [15–17]. However, lobaplatin therapy-related hyperalgesia following laparoscopic colectomy, a potential for significant post-operative opioid requirement, has not been investigated in a clinical situation. The current study hypothesized that chemotherapy using a single intraperitoneal administration of lobaplatin is associated with increased early postoperative hyperalgesia, as evidenced by a higher post-operative opioid requirement and pain intensity for 72 h after a major surgery.

## MATERIALS AND METHODS

### Participants

Informed consent was obtained from 84 subjects after approval from the Institutional Review Board of Liaocheng People's Hospital. The subjects were recruited preoperatively after a decision to operate made by the Department of General Surgery, during the period January to March, 2017. All subjects presenting for elective laparoscopic transabdominal resection of rectal cancer were included in the data analysis. The inclusion criteria included an American Society of Anesthesiology physical status of I, II or III, age of 46-65 years, and a body mass index less than or equal to 30. The exclusion criteria included contraindications to post-operative patient-controlled analgesia (PCA), an allergy to sufentanil, an allergy to lobaplatin, history of sinus bradycardia or atrioventricular block, history of major psychiatric disorder, history of substance abuse, current opioid use and history of chronic pain. Before surgery, the subjects were instructed in the use of the PCA device, numerical rating scale ([NRS], 0 = no pain, and 10 = worst pain imaginable) and the functional activity score (FAS) for pain assessments. The study was registered at chictr.org ( ChiCTR-IOR-17010915 ).

### Randomization and blinding

After informed written consent was obtained, a computer-generated randomization table was used to assign the patients into Group L and Group C (n = 40 per group) by an independent anesthetist before the surgery. Before the end of the surgery, a nurse blinded to the study prepared the experimental drug in the preparing room (lobaplatin 100 mg diluted in 500 mL in Groups L or 5% glucose in 500 mL in Group C), and then transferred it to the operation room. After the abdomen was closed, a single i.p. injection of lobaplatin in Group L or 5% glucose in Group C was administered via a drainage tube, and then the drainage tube was clipped for 6 h by the surgeon. The surgeons, anesthetists, nurses and members involved in the acute pain services were blinded to the patient classification.

### Anesthesia

None of the patients had received any medication before the induction of anesthesia. At the start of the anesthesia, a peripheral venous access was established in the right upper extremity, and a five-lead electrocardiogram, blood pressure, and oxygen saturation were continuously monitored using an automated system (Philips IntelliVue MP50). All subjects received a standardized general anesthetic using propofol (2.0 mg/kg), fentanyl (3 μg/kg) and nondepolarizing muscle relaxation using cisatracurium (0.15 mg/kg) on induction; then, tracheal intubation was performed 3 min later. During the period of anesthesia, general anesthesia was maintained using inspired sevoflurane at 1.5-2.5% and an i.v. infusion of remifentanil at 1 μg/kg/h to maintain hemodynamic limits (both mean blood pressure and heart rate) between 20% less and 20% more than preoperative levels. An additional 0.03 mg/kg of cisatracurium was administrated every hour from induction up to approximately 1 h prior to the end of operation and a bolus of fentanyl (50 μg) was administered after each additional 30 min of anesthesia. No other analgesics were administered during surgery. The subjects were ventilated at a tidal volume of 10 mL/kg, with the aim of maintaining the end-tidal CO_2_ concentration at 30-35 mmHg.

### Post-operative analgesia

After surgery, all the patients were transferred to the post-anesthesia care unit. The time of recovery of consciousness was defined as time 0. For 72 h after time 0, all subjects were provided with an i.v. PCA pump (Gemstar, Hospira, Inc., Lake Forest, IL, USA) as the sole analgesic. The PCA pump was programmed to deliver a bolus dose of 1.6 μg of sufentanil (2 mL), with a lockout of 5 min, no background infusion and a 4 h limit of 24 μg of sufentanil (30 mL). The goal of PCA was to maintain the at rest ≤4 post-operatively. Any subject with inadequate analgesia during the 72-h assessment period had the PCA sufentanil bolus increased to 3.2 μg (4 mL). For patients with a poor response to sufentanil, a NRS score of >7 (or NRS with coughing [NRSc]), or the occurrence of an obvious sufentanil-associated adverse effect, supplemental rescue boluses of 1 g of intravenous paracetamol were administered. The patients were monitored for adverse effects. Hypotension or bradycardia was treated using ephedrine or atropine, respectively, while respiratory depression was treated using naloxone and oxygen. The total dose requirements (72 h) for PCA sufentanil were calculated.

### Post-operative recordings and pain assessments

The trial was designed only to reach the endpoint of consumption of i.v. PCA sufentanil during the first 72 h after surgery. The secondary outcome measures were the postoperative pain intensity scores, both at rest and with coughing (NRSc), and FAS. Objective pain intensity was assessed using NRS from 0 to 10, where 0 = no pain, 10 = the worst pain imaginable, both at rest and with coughing, at 4, 24, 48 and 72 h after the operation. The FAS is a subjective pain intensity assessment performed by doctors using A, B and C, such that A = not limited: functional activity not limited because of pain, B = mild to moderate limitation: functional activity mildly to moderately limited because of pain, C = severely restricted: functional activity severely limited because of pain. The FAS was also recorded at 4, 24, 48 and 72 h after the operation. The potential side effects of sufentanil were also recorded: nausea, vomiting, pruritus, respiratory depression (defined as a ventilatory frequency of less than eight breaths per minute) and dizziness. Subjects experiencing severe pruritus were offered 0.04-0.1 mg of i.v. naloxone. Any subject who experienced vomiting more than three times a day was treated using i.v. tropisetron (4 mg).

### Statistical analysis

Sample size was based on a power calculation, which showed that 35 subjects per group were necessary to achieve 80% power to detect a 20% difference in sufentanil requirement, assuming a significance level of 0.05 [18, 19]. Assuming a dropout rate of 20%, a further 14 subjects were recruited so that each study group would have at least 35 subjects for a complete dataset. Therefore, a sample size of 84 was chosen for adequate data collection.

The Kolmogorov-Smirnov test was used to assess the distribution of variables. The homogeneity of variance was determined using Levene's tests. Quantitative data are expressed as mean and standard deviation or median and inter-quartile range. Inter-group comparisons were performed using repeated-measures analysis of variance. Categorical data are expressed as frequency and percentage and were analyzed using chi-squared tests or Fisher's exact tests when appropriate. Probability values of P < 0.05 were considered statistically significant. Statistical analysis was performed using SPSS for Windows version 16.0 (SPSS Inc. Chicago, IL, USA).

## RESULTS

**Figure 1:**
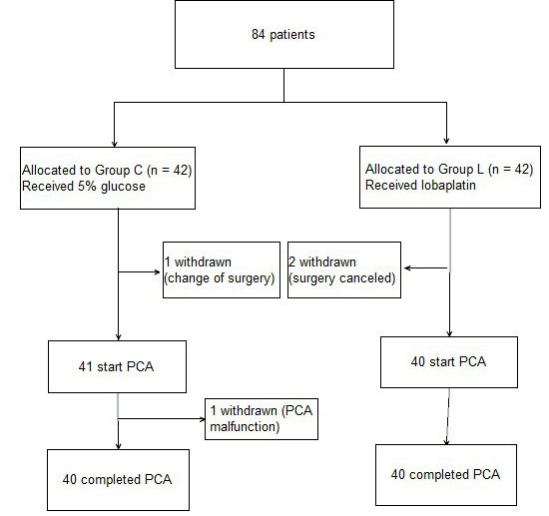
Patient flow chart Group C = control group; Group L = treated group. PCA, patient-controlled analgesia.

**Figure 2:**
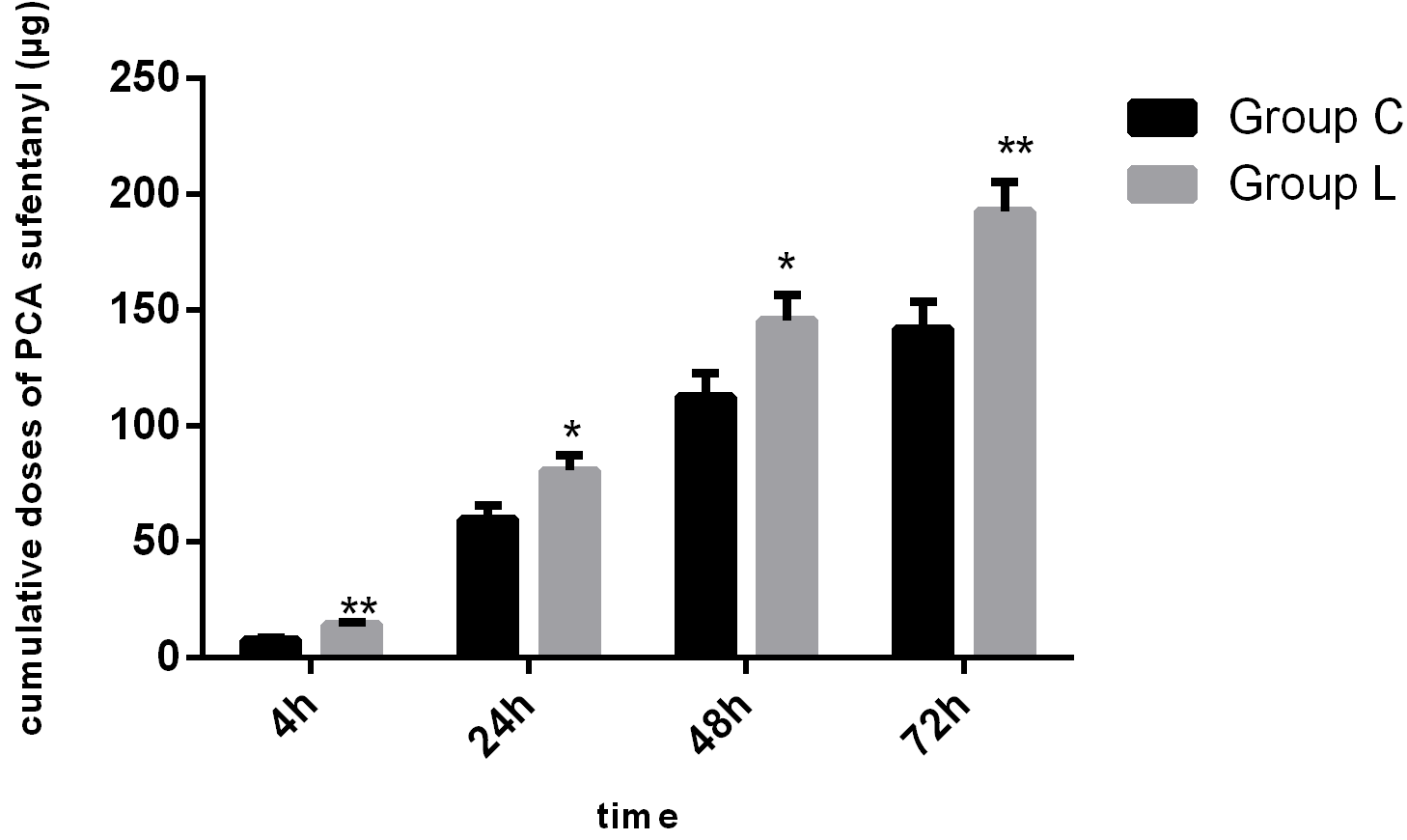
Sufentanil dosage during 72 h after surgery in Group C and Group L Continuous variables are presented as mean ± standard deviation. Group C = control group; Group L = treated group; *P < 0.05 *vs* Group C, **P < 0.01 *vs* Group C.

**Figure 3:**
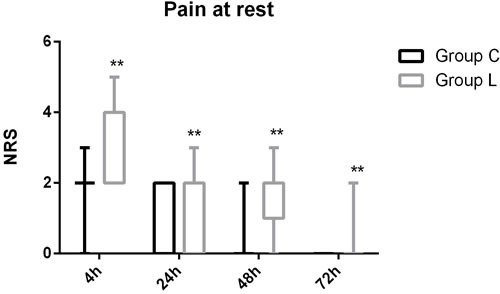
Pain score (NRS) at rest during 72 h after surgery in Group C and Group L Variables are presented as median (interquartile range). Group C = control group; Group L = treated group; *P < 0.05 *vs* Group C, **P < 0.01 *vs* Group C. NRS = numerical rating scale.

**Figure 4:**
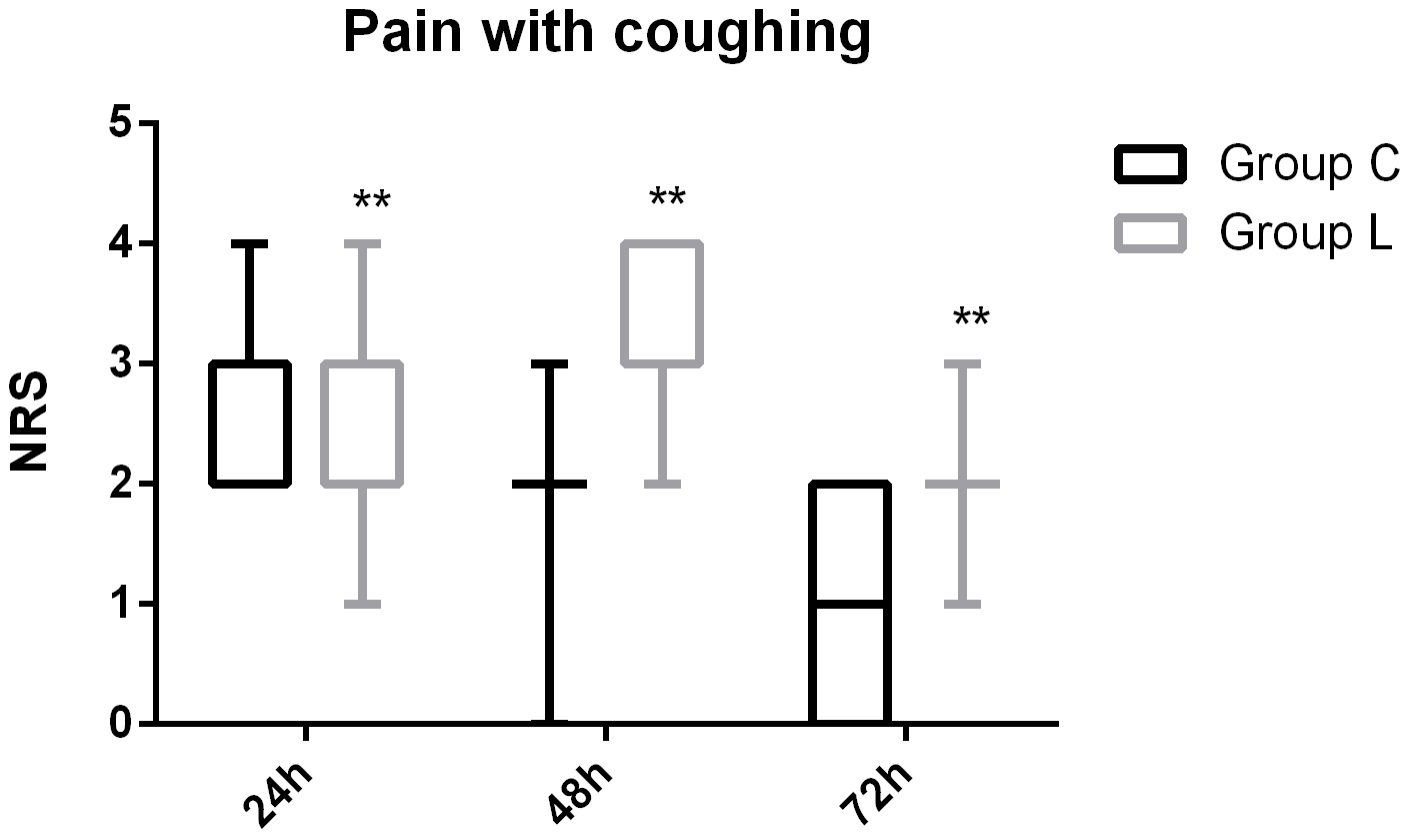
Pain score (NRS) with coughing during 72 h after surgery in Group C and Group L Variables are presented as median (interquartile range). Group C = control group; Group L = treated group; *P < 0.05 *vs* Group C, **P < 0.01 *vs* Group C. NRS = numerical rating scale.

The patient flow chart is presented in Figure 1. A total of 84 subjects were recruited. Four patients did not complete the study because of PCA malfunction, change of operation method, or surgery cancelation. Therefore, only data concerning the remaining 80 subjects are analyzed in this study. There were no significant differences in the demographic parameters of the two groups. There were also no differences in the duration of surgery and intra-operative medication requirements between the two groups (Table 1, P>0.05; Table 2, P>0.05). No subject required a change in analgesic technique. The total post-operative requirement of PCA sufentanil in Group L was significantly more than that in Group C (Figure 2, P < 0.05). The NRS pain intensity scores were low in both groups, but the pain intensity scores in the subjects of Group L were significantly higher at rest at 4h, 24h, 48h and 72h (Figure 3, P < 0.05) and with coughing at 24h, 48h and 72h (Figure 4, P < 0.05). There were significant differences in the FAS between Group L and Group C at 4 h (Table 3, P < 0.01) and 24 h (Table 3, P < 0.01), but no significant differences between the two groups at 48 h (Table 3, P>0.05) and 72 h (Table 3, P>0.05). Side-effects are presented in Table 4. There were no significant differences in the post-operative incidence of nausea and vomiting (Table 4, P>0.05) at 0-72 h between the two groups. There were also no differences in the incidence of pruritus and dizziness between the two groups (Table 4, P>0.05). No subject experienced respiratory depression in this study (Table 4, P>0.05). Groups C and L had similar rescue analgesia requirements (Table 5, P>0.05).

**Table 1:** Demographic and perioperative data

	Group C (*n* = 40)	Group L (*n* = 40)	*P*-value
Age (yr)	57.2 ± 5.5	57.6 ± 6.0	0.741
Male/female (n)	21/19	18/22	0.502
Body weight (kg)	67.0 ± 6.4	66.0 ± 8.1	0.569
BMI (kg/m^2^)	23.9 ± 1.5	23.8 ± 2.0	0.906
ASA: I/II/III (n)	3/32/5	3/33/4	1.00
Blood loss (mL)	138 ± 33	134 ± 29	0.576
Length of surgery (min)	169 ± 37	167 ± 45	0.825
Fluid infusion (mL)	1468 ± 208	1443 ± 273	0.646
Urine (mL)	415 ± 124	442 ± 139	0.376

Data are presented as mean ± standard deviation or number of patients (%); Group C = control group; Group L = treated group; no significant differences were observed (*P* > 0.05). BMI: Body Mass Index; ASA: American Society of Anesthesiology

**Table 2:** Anesthetic agents and intraoperative medication

	Group C (*n* = 40)	Group L (*n* = 40)	*P*-value
Propofol (mg)	134 ± 13	132 ± 16	0.569
Fentanyl (mg)	0.3 ± 0.1	0.3 ± 0.1	0.343
Cisatracurium (mg)	23.7 ± 3.0	22.5 ± 2.6	0.062
Sevoflurane (%)	2.5 ± 0.4	2.4 ± 0.3	0.623
Remifentanyl (mg)	0.7 ± 0.2	0.7 ± 0.2	0.976
Atropine (mg)	6 (15%)	4 (10%)	0.737
Ephedrine (mg)	8 (20%)	6 (15%)	0.556

Data are presented as mean ± standard deviation or number of patients (%); Group C = control group; Group L = treated group; no significant differences were observed (*P* > 0.05).

**Table 3:** Functional activity score

		Group C	Group L	*P*-values
FAS: C/B/A (n)	4 h	26/8/6	39/0/1	<0.001**
	24 h	0/1/39	0/9/31	0.007**
	48 h	0/0/40	0/3/37	0.120
	72 h	0/0/40	0/2/38	0.494

Data are presented as number of patients (%); Group C = control group; Group L = treated group; *P < 0.05 *vs* Group C, **P < 0.01 *vs* Group C.A = not limited: functional activity not limited because of pain, B = mild to moderate limitation: functional activity mildly to moderately limited because of pain, C = severely restricted: functional activity severely limited because of pain.

**Table 4:** Side effects

	Group C (*n* = 40)	Group L (*n* = 40)	*P*-value
Nausea, n(%)	7 (17.5)	12 (30)	0.189
Vomiting, n(%)	3 (7.5)	7 (17.5)	0.311
Pruritus, n(%)	2 (5)	6 (15)	0.263
Respiratory depression, n(%)	0 (0)	0 (0)	1.000
Dizziness, n(%)	3 (7.5)	5 (12.5)	0.712

Data are presented as number of patients (%); Group C = control group; Group L = treated group; no significant differences were observed (*P* > 0.05).

**Table 5:** Rescue analgesia

	Group C (*n* = 40)	Group L (*n* = 40)	*P*-value
n (%)	2 (5)	7 (17.5)	0.154

Data are presented as mean ± standard deviation or number of patients (%); Group C = control group; Group L = treated group; no significant differences were observed (*P* > 0.05).

## DISCUSSION

The current study is the first clinical study that describes increased post-operative opioid requirements and pain intensity in subjects following intraperitoneal chemotherapy with lobaplatin, with no significant difference in the incidence post-operative nausea and vomiting, pruritus, respiratory depression and dizziness.

CIPN is a potentially dose-limiting side-effect, commonly observed after application of various chemotherapeutic drugs including taxanes, vinca alkaloids, platinum compounds, bortezomib and thalidomide [2,3]. It is painful and/or disabling, causing a significant loss of functional abilities and decreasing quality of life reversibly or permanently. To date, many preventive and treatment strategies have been explored without significant efficacy. Most patients develop symptoms during, or soon after, chemotherapy administration, and in some patients it is associated with the first dose [20]. IPC has been demonstrated to be effective in clinical studies, and it could prolong overall survival in colorectal PC patients [21]. A prospective pilot study found that abdominal pain was the most common secondary effect in patients following IPC [22]. Moreover, some studies have demonstrated increased pain intensity early after IPC [23–25]. It has also been reported that 45% of patients undergoing IPC for PC using oxaliplatin displayed significant pain and limitations in terms of physical function after the treatment [26]. C. Schmidt and M. Creutzenberg retrospectively reviewed 78 patients, and found that postoperative consumption of epidural ropivacaine was a median of 5.0 g (0.8-11.2 g), and for piritramide a median of 210 mg (105-639 mg), after cytoreductive surgery using hyperthermic IPC [27]. Previous animal studies have revealed that a single intraperitoneal administration of oxaliplatin could decrease the mechanical threshold and induce mechanical allodynia, and the decreased threshold remained for 7 d after the treatment [28, 29].

This is consistent with the findings of the current study, in which an increased requirement for postoperative opioids was observed among subjects following IPC. The exact underlying mechanisms of some anti-cancer drugs have only recently begun to be understood. Dorsal root ganglia are considered to be the primary target of Pt drugs, where they cause apoptosis of sensory neurons and DNA damage [30]. The mechanisms underlying hyperalgesia following a single intraperitoneal administration of chemotherapeutic may be as follows. (1) The sensitization of Aβ - and C-fibers may contribute to the development of hyperalgesia induced by anticancer drugs, as demonstrated in rats [31]. (2) The concentration of anticancer drugs in the cerebrospinal fluid increases gradually after a single intraperitoneal administration, which may significantly increase the field potentials in the dorsal horn, eventually inducing hyperalgesia [32]. (3) Kim HK reported that oxidative stress and inflammatory processes might be involved in the development of CIPN through animal studies [33, 34]. (4) Another reason for hyperalgesia is that persistent visceral pain is always accompanied by hyperalgesia of the skin and internal organs [35].

Despite the strong evidence for chemotherapy-induced mechanical hyperalgesia, clinical prospective studies to date have yielded varying analgesic requirement patterns. The main reason for this is that human studies rely on the subjective responses of patients to assess pain. Moreover, humans have more control in experimental pain studies, compared with experimental animals in animal studies, and can choose not to participate at all or withdraw at any point during the study. In consideration of the subjectivity of NRS, we used FAS, a three-level scoring method recommended by the Victorian Quality Council to assess pain intensity, as an objective pain intensity assessment method. The FAS is a reliable and valid method, and is suitable for assessing dynamic pain in postoperative patients. It was observed that there were significant differences in the FAS between Group L and Group C at 4 h and 24 h, but no significant difference between Group L and Group C at 48 h and 72 h, indicating that IPC using lobaplatin could induce hyperalgesia in cancer patients.

Nausea and vomiting are common complaints after general anesthesia, with a multifactorial etiology. The risk factors for increased post-operative nausea and vomiting include younger age, female gender, prior history of dizziness, a higher requirement for perioperative opioids, a longer duration of anesthesia and specific types of surgery, such as laparoscopic surgery [36]. The incidence of nausea and vomiting did not differ significantly between the two groups. In the current study, the sufentanil requirement was higher in subjects following IPC, but the incidence of nausea and vomiting was similar. The incidence of pruritus, respiratory depression and dizziness, all known as adverse effects of sufentanil, was not significantly different between Group C and Group L.

The current study has some limitations. Firstly, it is a clinical study which provides few clues about the mechanisms of chemotherapy-induced hyperalgesia and only describes an increased opioid requirement and pain severity. Secondly, pain is an objective sensory phenomenon, and its intensity is associated with age, gender, psychological status and life experiences. The current study does not consider those factors, which may have affected the results. Finally, the current study was performed at a single center. Investigations of more diverse populations from different centers and the use of different surgical techniques would furnish more conclusive results.

In conclusion, IPC using lobaplatin was found to be associated with increased sufentanil requirements, an increased NRS and a worse FAS within the initial 72 hours after elective laparoscopic transabdominal resection of rectal cancer, compared with subjects who were not treated using lobaplatin. Future studies could explore the mechanisms underlying IPC-mediated lobaplatin-induced hyperalgesia in a clinical setting. A longer term follow-up of patients following IPC, to compare rates of chronic post-surgical pain, would also be potentially beneficial.

## Abbreviations

CIPN: chemotherapy-induced peripheral neuropathy
IPC: Intraperitoneal chemotherapy
PC: peritoneal carcinomatosis
PCA: patient-controlled analgesia
ASA: American Society of Anesthesiology
NRS: numerical rating scale
FAS: functional activity score
